# SCD1 promotes lipid mobilization in subcutaneous white adipose tissue

**DOI:** 10.1194/jlr.RA120000869

**Published:** 2020-09-25

**Authors:** Ying Zou, Yi-Na Wang, Hong Ma, Zhi-Hui He, Yan Tang, Liang Guo, Yang Liu, Meng Ding, Shu-Wen Qian, Qi-Qun Tang

**Affiliations:** 1 Key Laboratory of Metabolism and Molecular Medicine, Ministry of Education, Department of Biochemistry and Molecular Biology of the School of Basic Medical Sciences, and Department of Endocrinology and Metabolism of Zhongshan Hospital, Fudan University, Shanghai Medical College, Shanghai, China; 2 Key Laboratory of Metabolism and Molecular Medicine, Ministry of Education, Institutes of Biomedical Sciences, Fudan University, Shanghai, China

**Keywords:** stearoyl-CoA desaturase-1, triacylglycerol, adipocytes, lipolysis, lipophagy, oleic acid, thermogenesis

## Abstract

Beiging of white adipose tissue (WAT) has beneficial effects on metabolism. Although it is known that beige adipocytes are active in lipid catabolism and thermogenesis, how they are regulated deserves more explorations. In this study, we demonstrate that stearoyl-CoA desaturase 1 (SCD1) in subcutaneous WAT (scWAT) responded to cold stimulation and was able to promote mobilization of triacylglycerol [TAG (triglyceride)]. In vitro studies showed that SCD1 promoted lipolysis in C3H10T1/2 white adipocytes. The lipolytic effect was contributed by one of SCD1’s products, oleic acid (OA). OA upregulated adipose TAG lipase and hormone-sensitive lipase expression. When SCD1 was overexpressed in the scWAT of mice, lipolysis was enhanced, and oxygen consumption and heat generation were increased. These effects were also demonstrated by the SCD1 knockdown experiments in mice. In conclusion, our study suggests that SCD1, known as an enzyme for lipid synthesis, plays a role in upregulating lipid mobilization through its desaturation product, OA.

Two different kinds of adipose tissues are generally considered to exist in humans and other mammals. White adipose tissue (WAT) stores energy in the form of triacylglycerols [TAGs (triglycerides)]; on the contrary, brown adipose tissue (BAT) dissipates energy due to the great number of mitochondria and the associated uncoupling protein 1 (UCP1), which dissipates electrochemical gradients to generate heat (nonshivering thermogenesis) instead of ATP synthesis (1, 2). For the last decades, a group of brown-like adipocytes in WAT has been shown to take on brown adipocyte-like characteristics; these are named beige or brite adipocytes (3). As beige adipocytes also contain a high level of UCP1, they have the ability to dissipate energy through nonshivering thermogenesis. Previous studies suggested that human adults possess a certain amount of beige adipocytes, which are induced upon stimuli such as cold and exercise. Moreover, the activation of beige adipocytes is negatively correlated with body mass index (4). These characteristics of beige adipocytes demonstrated that they are potential therapeutic targets for obesity, type 2 diabetes, and other metabolic diseases.

Cold exposure induces beige fat biogenesis in subcutaneous WAT (scWAT) depots, promotes energy expenditure, and decreases mouse body weight and fat mass (5). During cold exposure, the β3-adrenergic receptor (β3AR) is activated by catecholamines released from sympathetic nerves. Furthermore, lipases such as adipose TAG lipase (ATGL) and hormone-sensitive lipase (HSL) are activated, leading to lipolysis. Lipolysis produces FFAs. FFAs can be transported out of adipocytes to provide energy for other tissues through β-oxidation. They can also be reesterified to form TAG. Therefore, simultaneous reactions of TAG hydrolysis and resynthesis create a futile thermogenic energy-consuming cycle, contributing to energy consumption independently of UCP1. During the TAG hydrolysis and resynthesis cycle, a quantity of new smaller lipid droplets (LDs) may form and acquire adipocytes with a multilocular phenotype (6).

In addition to the hydrolytic function of lipases, autophagy/lipophagy is demonstrated to be involved in mobilization of LDs (7). Lipophagy is the degradation of highly select LDs in the autolysosome (8). When lipophagy occurs, nascent microtubule-associated protein 1 light chain 3 (LC3) is cleaved off to become a soluble form, LC3-I. LC3-I is modified with phosphatidylethanolamine and becomes a membrane-bound form, LC3-II. LC3-II is recruited to the inner and outer membranes of autophagosomes and is degraded along with the autophagosome contents after lysosomal fusion. Thus LC3-II can serve as a reliable marker for autophagy (9). While p62-SQSTM1, a marker of protein aggregates, was shown to directly bind ubiquitinated proteins and LC3 for degradation by autophagy, therefore the degradation of p62 indicated the activation of autophagy (10–13). During LD mobilization, lipolysis of lipases and lipophagy have been shown to be interrelated. Some researchers have reported that, in hepatocytes, ATGL moves through the PPARα/peroxisome proliferator-activated receptor coactivator 1α (PGC1α) pathway to regulate sirtuin 1, which further induces autophagy and FA oxidation (14, 15). Others found that LC3 is associated with ATGL-mediated lipolysis in BAT. ATGL was shown to have LC3-interacting region motifs. When a single LC3-interacting region motif was mutated, ATGL was displaced from the LD and lipolysis was disrupted (16). Thus, lipophagy and cytoplasmic lipases complement each other in lipolysis.

The changes in the lipidome of total scWAT in response to cold have been reported (5, 17), but the lipidome changes that focus on TAG of adipocytes have not been determined. Desaturases are enzymes responsible for modifying FA desaturation. It is reported that desaturases respond to cold regulation in WAT. Among those desaturates, stearoyl-CoA desaturase (SCD) is the most prominently regulated (17). However, the role of SCD in response to cold is unknown. SCD is a key regulator of de novo lipogenesis (18). It catalyzes the synthesis of MUFAs, palmitoleic acid (16:1n7), and oleic acid (OA; 18:1n9) from saturated FAs (SFAs), palmitic acid (16:0), and stearic acid (SA; 18:0), respectively. MUFAs are used to synthesize lipids, like TAGs, cholesterol esters, and phospholipids, which construct cellular membranes or act as lipid signaling molecules (19). Four SCD isoforms *(Scd1*–*Scd4*) have been identified in the mouse (20–23). *Scd1* and *Scd2* are expressed in lipogenic tissues, such as liver and adipose tissue; *Scd3* is mainly expressed in the skin, preputial gland, and Harderian gland; while *Scd4* is primarily expressed in the heart (20, 23, 24). The team of James M. Ntambi has reported that global SCD1-deficient mice (SCD1^−/−^) showed decreased hepatic TAG content, reduced body obesity, increased insulin sensitivity, and resistance to diet-induced weight gain (25, 26). The results may suggest the important role of SCD1 for de novo lipogenesis. However, adipose tissue-specific deletion of SCD1 driven by aP2-Cre transgene had no effect on glucose or insulin resistance or liver TAG accumulation (27). It is very intriguing that SCD1 KO mice showed hypothermia when exposed to 4°C (28). Another study reported that UCP1-deficient mice are unexpectedly cold sensitive. In UCP1-deficient mice, the increased lipolysis in scWAT might account for maintaining body temperature, which was associated with elevated SCD1 expression (29). These studies indicate a role of SCD1 in lipid mobilization and thermoregulation. However, the paradox of SCD1 for lipid synthesis and mobilization has not been investigated and solved.

Here, we found that the percentage of MUFAs and the expression of SCD1 were upregulated during cold exposure in adipocytes of mouse scWAT. Overexpression of SCD1 in mouse scWAT suggested that SCD1 can induce lipolysis through upregulating lipases and lipophagy pathways, further promoting fat mobilization and energy expenditure. In summary, we found that SCD1 plays an effective role in regulating adipocyte lipid mobilization through alteration of FA composition.

## MATERIALS AND METHODS

### Animals

The Fudan University Basic Medical College critiqued and approved the animal protocol (#20180302-010). We purchased 4- to 6-week-old male C57BL/6J mice from Nanjing University Model Animal Research Center. We used fatty acid-binding protein 4 (Fabp4)-bone morphogenetic protein 4 transgenic (BMP4 TG) mice and Fabp4-Cre-Bmp4^LoxP/LoxP^ (BMP4 KO) mice, which were used in our previous study (30). We housed the mice with enough food and water on a 12 h light/12 h dark cycle at 22°C. WT male mice aged 8–10 weeks were used unless otherwise specified. For cold acclimation, the mice were housed in a temperature-controlled room at 4°C for the indicated time. All of these animal experiments follow the National Institutes of Health *Guide for the Care and Use of Laboratory Animals*.

### Metabolic studies

We housed and monitored the mice individually for 48 h in metabolic cages (Oxymax-CLAMS Comprehensive Lab Animal Monitoring System, Columbus Instruments); during this period, regular chow and water were available ad libitum. The mice were housed at 22°C under a 12 h light and 12 h dark cycle. The first 24 h is used for mice to acclimate to the system. We measured the VO_2_ of the mice during the next 24 h. We injected the mice with CL-316,243, which is a β3AR agonist, to measure the acute respiratory response (VO_2_) of the mice during the next 24 h. The oxygen consumption of each mouse was monitored every 25 min for the entire 72 h. The oxygen consumption (in milliliters per kilogram per hour) and heat (in kilocalories per kilogram per hour) of each mouse were calculated according to its body weight. The light cycle was from 0700 to 1900, and the dark cycle was from 1900 to 0700.

### Cold tolerance test

To test the cold tolerance of the mice, we housed the mice in a temperature-controlled room (MMM Friocell, Germany) at 4°C. Rectal temperature was measured with a rectal probe (Physitemp, BAT-12) at the indicated time.

### Mature adipocytes and SVF isolation

We harvested and cut up mouse adipose tissues. Then, we digested them with 0.075% collagenase (Collagenase VIII; Sigma C2139) at 37°C for 30–45 min. We filtered the digested tissues through a 100 μm mesh filter and then centrifuged them at 240 *g* for 5 min. We removed the adipocytes to a new tube, washed them with PBS, and centrifuged them at 240 *g* for 5 min for collection. We used an ammonium chloride lysis buffer (1.5 M NH_4_Cl, 100 nM KHCO_3_, 10 nM Na_2_EDTA) to resuspend the cellular pellets, which contains stromal vascular fractions (SVFs). After the centrifugation, we collected the SVFs for examination.

### Cell culture assay experiments

C3H10T1/2 mesenchymal stem cells were donated by Dr. M. Daniel Lane from Johns Hopkins University, and we tested for mycoplasma before the experiments. The cells were propagated and maintained at 37°C in a 5% CO_2_ environment in DMEM, which is supplemented with 1% penicillin and 1% streptomycin. Cells were cultured in 10% (v/v) calf serum at a low density. Two days after the cells reached confluence, the day was designated as 0 (referred to day 0). Preadipocytes were differentiated into adipocytes with DMEM, which contains 10% (v/v) FBS (Invitrogen), 1 μM rosiglitazone, 0.5 mM 3-isobutyl-1-methylxanthine, 1 μg/ml insulin, and 1 μM dexamethasone, for 2 days. The cells were then cultured in DMEM, which was supplemented with 10% (v/v) FBS, 1 μM rosiglitazone, and 1 μg/ml insulin, for another 2 days. From day 4, the adipocytes were fed with DMEM, which contained 10% (v/v) FBS. DMEM was replaced every other day until the adipocytes were used for experiments on day 6(if not specified).

### Construction of adenoviral expression vectors and infection

We used the ViraPower adenoviral expression system (Invitrogen, Carlsbad, CA) to generate recombinant adenovirus for SCD1 overexpression (Ad-SCD1), and we used LacZ recombinant adenovirus (Ad-LacZ) as the negative control. We used pBlock-it (Invitrogen) adenoviral expression vectors, which encoded the shRNA of mouse SCD1 or LacZ, to construct Ad-shSCD1 or Ad-shLacZ according to the manufacturer’s protocol. The sequences for shLacZ or shSCD1 (5′ to 3′) were: shLacZ, CTACACAAATCAGCGATTT and shSCD1, CACCGAGTTTCTAAGGCTACTGTCTTCGAAAAGACAGTAGCCTTAGAAAC. We amplified and purified adenovirus vectors with Sartorius adenovirus purification kits (Sartorius, Göttingen, Germany). We diluted 20 μl of purified adenovirus (concentration 1 × 10^11^ PFU/ml) with 180 μl of sterile 1× PBS. We injected 100 μl of adenovirus solution to both sides of scWAT for each mouse twice a week. Two days after the last injection, the mice were subjected to further studies.

We described the C3H10T1/2 adipocyte differentiation method in the Cell culture assay experiments section. On day 4, we infected the adipocytes (in 3.5 cm dishes) with 5,000 PFU of adenovirus (Ad-shLacZ or Ad-shSCD1, Ad-LacZ or Ad-SCD1) supplemented with 8 μg/ml of polybrene (Sigma) to enhance adenovirus infection efficiency. After 24 h, we changed the supernatant to DMEM containing 10% (v/v) FBS for another 2 days.

### Oil Red O staining

We rinsed differentiated adipocytes with sterile 1× PBS twice and used 10% buffered formalin at room temperature (RT) for 20 min to fix them. Afterwards, we stained the adipocytes with freshly made Oil Red O for 120 min at RT. Then, we removed the Oil Red O solution and washed the cells three times with distilled water. We observed the stained LDs with light microscopy and photographed them.

### Glycerol release assay

We pretreated differentiated C3H10T1/2 adipocytes with adenovirus for a lipolysis assay at the indicated time. On day 7, the adipocytes were cultured in serum-free DMEM containing 2% BSA (Sigma) for the basal lipolysis assay or with 0.1 μM CL-316,243 for the stimulated lipolysis assay. We used a glycerol release kit (Applygen, E1002) to measure glycerol content in the culture medium according to the manufacturer’s protocol. Glycerol levels were normalized with total cellular protein or Oil Red O quantification.

### Immunofluorescence microscopy

We seeded C3H10T1/2 cells on coverslips and fixed them with 4% polyformaldehyde for 10 min at RT. Then, we washed the cells with sterile 1× PBS three times. Next, we used 0.2% Triton X-100 in sterile 1× PBS to permeabilize the cells for 10 min at RT. We blocked the cells with 1% BSA in sterile 1× PBS for 1 h at RT and incubated the cells with appropriate primary and secondary antibodies in 1% BSA. To visualize LDs, we added 0.3 μg/ml BODIPY 493/503 dye (Thermo Fisher, D3922) to the cells during incubation with secondary antibodies and then stained cells with DAPI (Sigma). We mounted cell samples on glass slides with Vectashield antifade mounting medium and scanned the samples on a Leica SP5 confocal system, which was mounted on an inverted microscope.

### RNA extraction and quantitative real-time PCR

We harvested tissues or cells and used 1 ml of TRIzol reagent (Invitrogen) to lyse them. Then, we used RevertAid First Strand cDNA Synthesis kit (Thermo Fisher, K1622) to reverse transcribe 1 μg of total RNA. We used 2× PCR Master Mix (Power SYBR Green; Applied Biosystems) to perform quantitative PCR (qPCR) using the 7500 Fast Real-Time PCR System (Applied Biosystems). We used the ΔΔCt method to calculate all gene expression and used 18s rRNA as an endogenous control. We set the average of the control group as one and represented all the results as the relative mRNA expression. Data was gathered from different individual mice. Primers that were used in the present study are listed in supplemental Table S1.

### Western blotting

We lysed mouse tissues or cells in 2% SDS)buffer supplemented with phosphatase and protease inhibitors (Roche). We used a BCA assay (Pierce, Thermo Fisher) to test total protein concentration. As a measure of autophagic flow, Western blots for LC3 and p62 were performed in cells treated with the lysosomal inhibitor, leupeptin (Selleck;10 μM) for 12 h. We loaded 30 μg of protein to SDS-PAGE, and subsequently transferred the protein onto 0.22 μm PVDF membranes. We used 5% BSA to block membranes and then incubated the membranes with primary antibodies and respective secondary antibodies and subjected them to electrochemiluminescence.

The primary antibodies used in this experiment were: p-HSL^ser660^ (1:1,000; CST, CST4139), total-HSL (1:1,000; CST, CST4107), HSP90 (1:1,000; Santa Cruz, sc7947), ATGL (1:1,000; CST, CST2138), SCD1 (1:1,000; CST, CST2794), p62 (1:1,000; MBL, PM045), LC3 (1:1,000; MBL, M186-3), FA desaturase-1 [FADS1 (delta-5-desaturase)] (1:1,000; Abcam, ab126706), FA desaturase-2 [FADS2 (delta-6-desaturase)] (1:1,000; Abcam, ab232898).

### Luciferase reporter assay

We cloned the fragments of SCD1 (NM_009127.4) promoter into pGL3-Basic vector to generate promoter-reporter constructs. We verified all constructs by sequencing. We seeded 293T cells in 24-well plates. Then, we transiently transfected 293T cells with plasmids, which contained firefly luciferase reporters, recombinant promoter-reporter constructs, and different doses of Smad1-pCMV plasmid. After incubation for 48 h, we used the Dual-Luciferase Reporter Assay kit (Promega) to measure the luciferase activity. We used Renilla luciferase activity to normalize the transfection efficiency.

### Lipid extraction, derivatization, and FAME analysis

We used a modified Folch method for extracting lipids (31). We homogenized adipocyte samples with 1 ml of chloroform:methanol (2:1, v/v), which contained 1 mg/10 ml butylated hydroxytoluene. Next, we added 340 μl of acidified saline (0.01 N HCl, 0.9% NaCl). We vortexed the samples for 2 min and centrifuged at 16,000 *g* at RT for 20 min to isolate the organic layer. We used nitrogen gas to dry the organic layer and subjected it to a heptane/isopropyl ether/glacial acetic acid (60/40/3, v/v/v) mixture for TLC on silica gel-60 plates. The TAG bands dyed with rhodamine B (32) were scraped and extracted with 700 μl of chloroform. Then, the liquid was transferred to new glass tubes with screw caps and dried under a nitrogen stream. Next, we added 150 μl of 2 M KOH at 60°C for 30 min under a nitrogen atmosphere to hydrolyze TAG. After cooling to RT, we added 700 μl of 50 mM phosphate buffer (pH 7.4) and 300 μl of 2.5 M HCl. We added 2 ml of hexane:ethyl ether (1:1, v/v) for extraction, removed the upper layer, and dried under nitrogen flow.

### GC-MS analysis of FA composition

We used a previously reported method (33, 34) with some modifications to methylate FA. In short, we added methyl tricosanoate and butylated hydroxytoluene to a Pyrex tube and then added the samples and the methanol-hexane mixture (as internal standard). We cooled down the tubes by liquid nitrogen and added precooled acetyl chloride. We screw-capped the tubes and kept them in the dark for 24 h at RT. We used K_2_CO_3_ solution to neutralize the resultant mixture and hexane to extract methylated FAs. After resting, we transferred the top layer into a glass sample vial. The extraction process was repeated two times and then the combined supernatants were evaporated. We used hexane to redissolve the resultant residues and analyzed with GC-MS analysis. Methylated FAs were confirmed and quantified by their mass spectral data. The molar percentages were calculated for SFAs, unsaturated FAs (UFAs), MUFAs, and PUFAs, respectively.

### Oxygen consumption rate of scWAT

We used an Oxygen Meter (Strathkelvin Instruments), which contains a Mitocell (MT200) mixing chamber, to measure the oxygen consumption rate (OCR) of scWAT. We cut approximately 100 mg of scWAT tissue into small pieces and then tested for OCR. We recorded the oxygen concentration for 2 min and used 782 Oxygen System version 4.0 software (Strathkelvin Instruments) to calculate the OCR.

### Statistical analyses

We expressed all results as mean ± SD. We used the unpaired Student’s *t*-test to analyze differences between the two groups and ANOVA for multiple groups. We performed statistical analysis and constructed graphs using Prism 6. *P* < 0.05 was considered statistically significant.

## RESULTS

### The percentage of MUFAs, especially OA (C18:1) of TAG, in scWAT adipocytes is increased after cold exposure

**Fig. 1.**
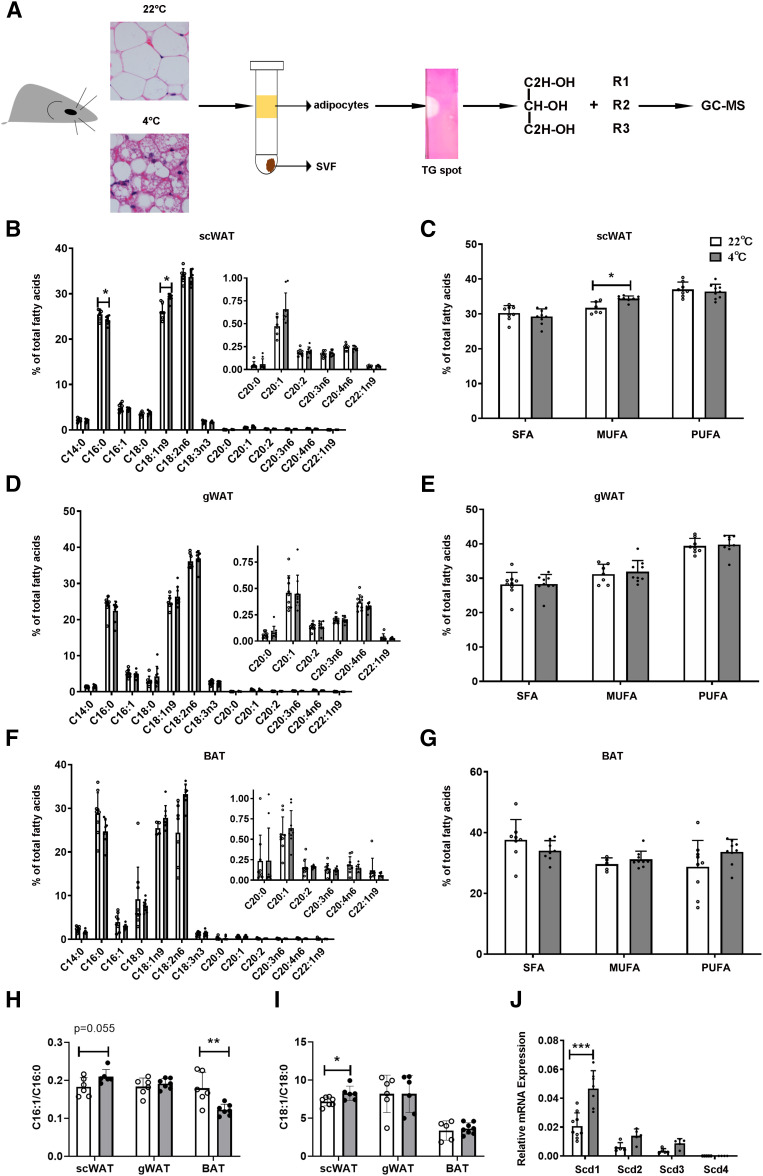
The changes of FAs in the TAG of WAT and BAT adipocytes in response to cold exposure. A: Schematic model describes the method of TAG FA in adipocyte analysis. Adipocytes were enzymatically dissociated from scWAT, gWAT, and BAT of C57BL/6J mice either at RT (22°C) or during cold exposure (4°C) for 3 days. TAGs were separated and collected by TLC. After being hydrolyzed by KOH, lipids were esterified to form methyl esters. Methyl esters were detected by GC-MS. Lipid species were quantified based on comprehensive peak area. B, C: FAs were obtained from scWAT of mice housed at RT (22°C) or during cold exposure (4°C) for 3 days. (n = 8 for each group) according the methods in A. FA profiles were analyzed (B) and percentage of SFAs, MUFAs, and PUFAs were calculated (C). D, E: The same analysis as in B and C from gWAT samples of the same mice. F, G: The same analysis as in B and C from BAT samples of the same mice. H, I: SCD desaturation indices were calculated accordingly: SCD-16 = 16:1/16:0 and SCD-18 = 18:1/18:0, as shown in H and I, respectively. J: Relative mRNA expression of *Scd1*–*Scd4* in scWAT from mice housed at RT or during cold exposure for 3 days. (n = 7–9 for each group). Statistical analysis: unpaired Student’s *t*-test in B–J. Data were expressed as mean ± SD. *P < 0.05, **P < 0.01, ***P < 0.001.

Adipocytes store energy in the form of TAG, which is ready to mobilize at the time of energy demands. To investigate the adaptive changes of TAG lipid composition in adipocytes after cold exposure (4°C), we isolated WAT and BAT adipocytes from RT- and cold exposure-treated mice by enzymatic dissociation and then separated TAG on TLC plates and hydrolyzed TAG by KOH. Next, FAs from TAG were esterified to form methyl esters and analyzed by GC-MS (**Fig. 1A**). Our results indicated that, in mouse scWAT adipocytes, the percentage of total MUFAs, especially OA (C18:1), was significantly increased after cold exposure (Fig. 1B, C). However, in mouse gonadal WAT (gWAT) and BAT adipocytes, the percentage of total MUFAs in TAG increased but showed no significant difference between RT and cold exposure (Fig. 1D–G). These results indicate that cold exposure increases the MUFA percentage of TAG in scWAT adipocytes and induces lipid remodeling in TAG.

SCD is the main enzyme responsible for the desaturation of SFAs to MUFAs (35). We used these FA values to calculate SCD desaturation indices [i.e., SCD-16 (C16:1/C16:0); SCD-18 (C18:1/C18:0)]. It was notable that the SCD desaturation indices were most strongly increased after cold exposure in scWAT adipocytes, which was consistent with the increase of the MUFA ratio of this tissue (Fig. 1H, I). There are four *Scd* isoforms in the mouse genome. To specify the isoforms in scWAT in response to cold, we measured *Scd1*–*Scd4* mRNA levels in scWAT adipocytes from RT- treated (22°C) and cold-treated (4°C) mice. Compared with *Scd2*, -*3*, and -*4*, we found, in scWAT adipocytes, that the *Scd1* mRNA level was highest at RT (22°C), and its expression increased the most after cold exposure (4°C) (Fig. 1J). Generally, these data suggested that cold exposure caused an increased MUFA proportion of TAG in scWAT adipocytes and that SCD1 may be a candidate to mediate this process. So, we focused on exploring SCD1 function in adipose tissue.

### The expression of SCD1 is enriched in scWAT and BAT and is highly induced by cold exposure in scWAT adipocytes

**Fig. 2.**
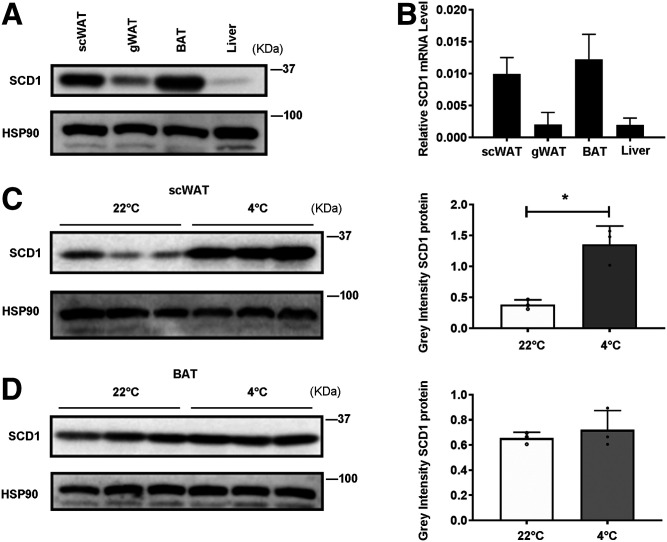
SCD1 is enriched in scWAT and BAT. It is highly induced by cold exposure in adipocytes. A: Tissue distribution of SCD1 protein expression in adipocytes of scWAT, gWAT, BAT, and liver from mice housed at RT (22°C). B: Tissue distribution of *Scd1* mRNA expression (relative to *18S rRNA*) in adipocytes of scWAT, gWAT, BAT, and liver from mice housed at RT. C: Western blot detection of SCD1 expression in scWAT adipocytes of mice at RT or during cold exposure (4°C) for 3 days (left panel), and the gray density of those bands (right panel) (n = 3 for each group). D: Western blot detection of SCD1 expression in BAT adipocytes of mice at RT or during cold exposure for 3 days (left panel), and the gray density of those bands (right panel) (n = 3 for each group). Statistical analysis: unpaired Student’s *t*-test in C and D. Data were expressed as mean ± SD. *P < 0.05, **P < 0.01, ***P < 0.001.

First, we examined the expression levels of SCD1 in three adipose tissues and liver from mice housed at RT (**Fig. 2A, B**). Consistent with previous findings (29), SCD1 protein and mRNA expression were significantly higher in scWAT and BAT compared with gWAT and liver, which was confirmed by Western blot analyses (Fig. 2A) and real time-qPCR, respectively (Fig. 2B). We also detected FADS1 and FADS2, which are the rate limiting enzymes for the biosynthesis of long-chain PUFAs in adipose tissues and liver. We found that, unlike SCD1, FADS1 and FADS2 are enriched in liver (supplemental Fig. S1A). To determine the changes of SCD1, FADS1, and FADS2 in scWAT and BAT adipocytes in response to cold, we isolated scWAT and BAT adipocytes from RT- and cold-treated mice and analyzed SCD1, FADS1, and FADS2 protein levels. We observed a significant induction of SCD1 expression in adipocytes of scWAT but not in BAT after cold exposure (Fig. 2C, D), while FADS1 and FADS2 protein levels did not change after cold exposure (supplemental Fig. S1B, C). Adipose tissue is composed of mature adipocytes and SVF. The latter includes preadipocytes, red blood cells, and various immune cells (eosinophils, macrophages, mast cells, and T cells) (36, 37). To determine whether SCD1 expression is restricted to adipocytes but not SVFs in scWAT after cold exposure, we isolated scWAT adipocytes and SVF from RT- and cold-treated mice. We found that SCD1 was more abundant in mature adipocytes than SVF (supplemental Fig. S2A). Consistent with Fig. 2C, after cold exposure, *Scd1* mRNA expression was also induced in mature adipocytes but not in SVF (supplemental Fig. S2B). Collectively, SCD1 is highly expressed in scWAT and BAT, with its expression significantly induced in scWAT adipocytes by cold exposure. These data indicated that SCD1 plays an important role in scWAT lipid metabolism in response to cold, which prompts us to investigate this hypothesis.

### SCD1 promotes lipolysis in cultured adipocytes

**Fig. 3.**
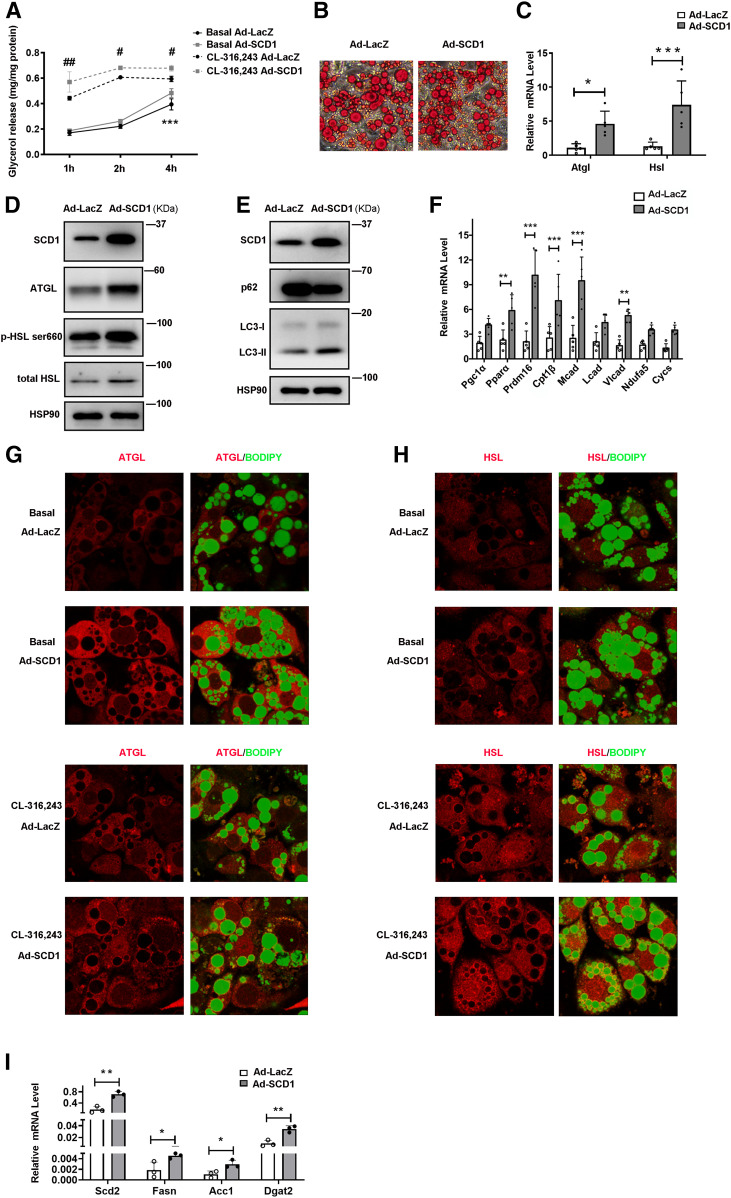
SCD1 promotes lipid mobilization in cultured adipocytes by upregulating levels of lipases, lipophagy, and lipogenesis. C3H10T1/2 mesenchymal stem cells were differentiated for 6 days during the adipogenic program. A: Relative glycerol release from C3H10T1/2 adipocytes treated with Ad-LacZ or Ad-SCD1 for 3 days before the lipolysis experiment. Adipocytes were cultured without or with 0.1 μM CL-316,243 (cell culture medium was changed to phenol red-free medium containing 2% BSA without FAs). The glycerol content of culture medium was quantified with a glycerol release kit (Applygen, E1002) at 1, 2, and 4 h (n = 4 for each group). Total cellular protein was quantified for the glycerol normalization. Experiments were independently repeated three times. B: Oil Red O staining of C3H10T1/2 cells treated with Ad-LacZ or Ad-SCD1. C: Relative mRNA expression of *Atgl* and *Hsl* in cells treated with Ad-LacZ or Ad-SCD1 for 3 days (n = 5 in each group). D: Representative Western blot of the lipases (ATGL, total HSL, and HSL phosphorylation) in adipocytes. E: Representative Western blot of the lipophagy markers protein (p62 and LC3-II) in cells treated with Ad-LacZ or Ad-SCD1 for 3 days. F: Relative mRNA expression of the transcriptional regulators and FA oxidation enzymes, nuclear-encoded mitochondrial genes in cells treated with Ad-LacZ or Ad-SCD1 for 3 days (n = 5 in each group). G, H: Immunofluorescence staining with anti-ATGL antibodies (G) and anti-HSL antibodies (H) was performed to reveal localization of endogenous ATGL or HSL in C3H10T1/2 adipocytes treated with Ad-LacZ or Ad-SCD1 for 3 days under basal or 0.1 μM CL-316,243-stimulated 20 min state. LDs were stained with BODIPY 493/503. I: Relative mRNA expression of the lipogenesis genes *Scd2*, *Fasn*, *Dgat2*, and *Acc1* in cells treated with Ad-LacZ or Ad-SCD1 for 3 days (n = 3 in each group). Statistical analysis: two-way ANOVA in A, unpaired Student’s *t*-test in C, F, and I. Data were expressed as mean ± SD. *P indicated for the comparisons at the basal condition (Ad-LacZ vs. Ad-SCD1); #*P* indicated for the comparisons at the CL-316,243 stimulated condition (Ad-LacZ vs. Ad-SCD1). Data were expressed as mean ± SD. *P < 0.05, **P < 0.01, ***P < 0.001. #*P* < 0.05, ##*P* < 0.01, ###*P* < 0.001.

To further explore the role of SCD1 in mature adipocytes, we used the C3H10T1/2 adipocyte model in vitro, which is the classic model for studying adipocyte browning (30, 36). We infected adipocytes with recombinant adenovirus Ad-SCD1, with Ad-LacZ as a control, to examine the effect of SCD1 overexpression on lipid mobilization. As expected, SCD1 protein level was effectively increased by infecting the cells with Ad-SCD1 (**Fig. 3D**). In differentiated adipocytes, SCD1 significantly enhanced glycerol release both under basal conditions and after the treatment with CL-316,243 (a β3AR agonist) (Fig. 3A). Moreover, SCD1 overexpression in differentiated adipocytes led to smaller LD size than the control group, as illustrated by Oil Red O staining (Fig. 3B). We also treated mature adipocytes with LDs well-developed (C3H10T1/2 cell differentiated on day 8) with adenovirus Ad-SCD1 or Ad-LacZ for 3 days and performed Oil Red O staining on day 11 to further confirm that smaller LDs are due to SCD1 increased lipolysis. As shown in supplemental Fig. S3A, SCD1 overexpression on day 11 of differentiated adipocytes also led to smaller LD size than the control group, as illustrated by Oil Red O staining. As evidenced by the decreased content of lipid and the increased release of glycerol in C3H10T1/2 adipocytes overexpressing SCD1, it is suggested that SCD1 may promote lipolysis in adipocytes. To study the effect of SCD1 on promoting lipolysis, we examined lipases and lipophagy-related factors. In C3H10T1/2 adipocytes overexpressing SCD1, we found significantly increased expression of ATGL and HSL at mRNA as well as protein levels (Fig. 3C, D) and enhanced phosphorylation of HSL at Ser660 (Fig. 3D). To determine whether lipases are induced by SCD1 overexpression in the situation of β3AR activation, we added CL-316,243 to C3H10T1/2 adipocytes infected with Ad-SCD1 or Ad-LacZ and tested lipase protein levels. Lipases in SCD1 overexpression cells indeed upregulated at basal conditions and were further stimulated by CL-316,243 (supplemental Fig. S3B). These data demonstrated that SCD1 promotes lipase expression and lipolysis in both basal and β3AR-activated situations. The degree of accumulation of LC3-II after lysosomal inhibition reflects LC3-II flux or autophagy flux, which is in line with the autophagy research guidelines (38). In adipocytes with leupeptin, SCD1 overexpression increased LC3-II flux but decreased p62 when compared with the control Ad-LacZ (Fig. 3E), suggesting that SCD1 promoted lipophagy. We also examined the FA oxidation enzyme genes, transcriptional regulator genes, and nuclear-encoded mitochondrial genes in adipocytes. The transcriptional regulator genes, including *Pparα* and PRD1-BF1-RIZ1 homologous domain containing 16 (*Prdm16*), and FA oxidation genes, *Cpt1β*, *Mcad*, and* Vlcad*, were significantly upregulated by the overexpression of SCD1 (Fig. 3F).

It has been reported that the lipase activity of ATGL and HSL in vivo was determined by their LD localization. They translocated from cytoplasm to LDs to mediate hydrolysis of TAG (35, 39, 40). To analyze the effect of SCD1 on localization of endogenous ATGL and HSL, we carried out immunofluorescence microscopy in C3H10T1/2 adipocytes in the basal and stimulated state. In the basal state, high levels of ATGL and HSL were found in SCD1 overexpression adipocytes’ cytoplasm (Fig. 3G, H), consistent with Fig. 3C and D. In stimulated state, SCD1 overexpression promoted more ATGL and HSL surrounding LDs to mobilize TAG lipolysis (). These data suggested that SCD1 promoted lipolysis through upregulating lipases and enhancing lipophagy in adipocytes. Furthermore, upregulation of the lipogenesis genes *Scd2*, *Fasn*, *Dgat2*, and *Acc1* mRNA levels was detected in the C3H10T1/2 cells overexpressing SCD1 (Fig. 3I). In conclusion, these results suggested that SCD1 may play an important role in regulating lipid mobilization.

### Knockdown of SCD1 inhibits lipolysis in adipocytes

**Fig. 4.**
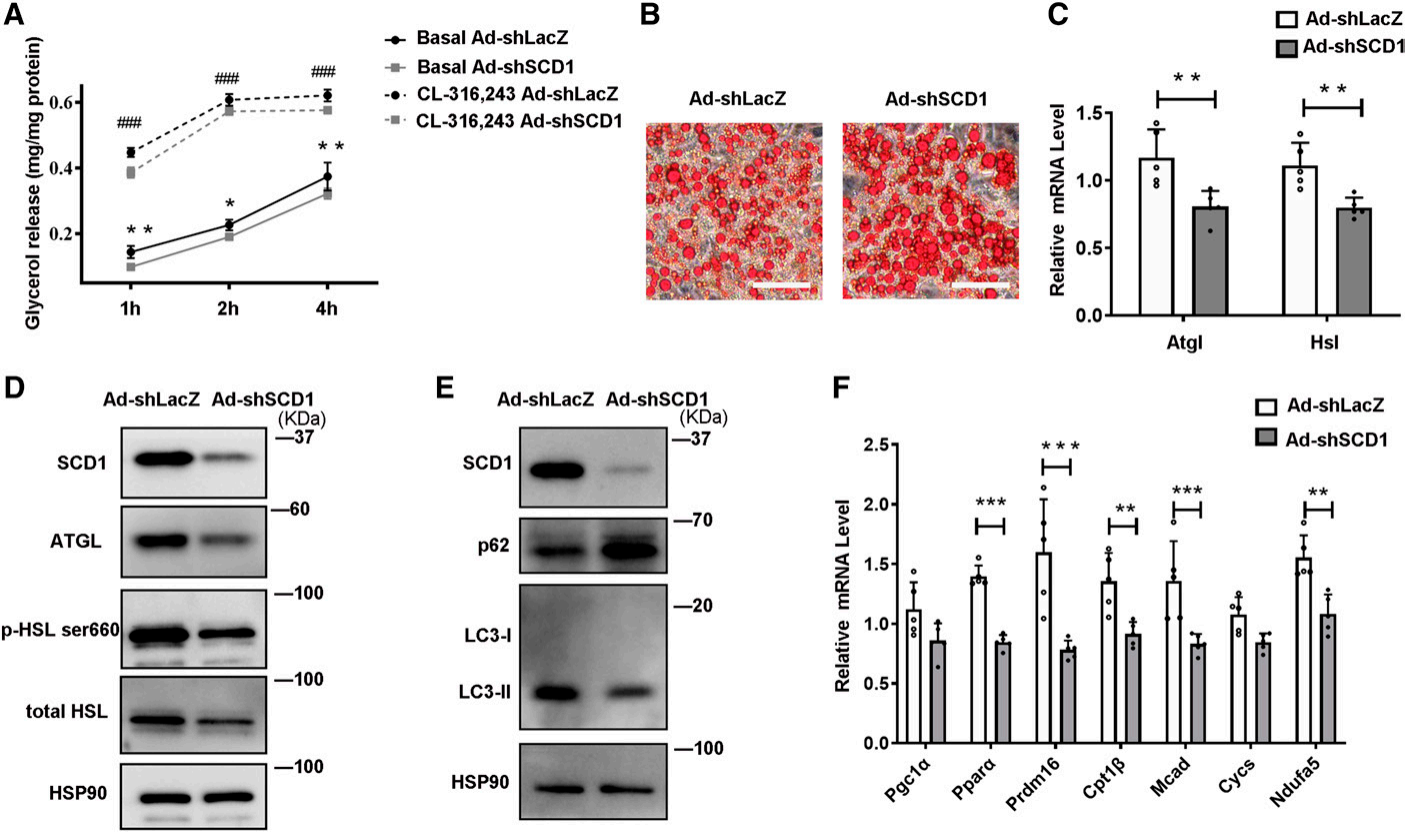
Knockdown SCD1 inhibits lipolysis in cultured adipocytes by downregulating lipases and lipophagy. C3H10T1/2 mesenchymal stem cells were differentiated for 6 days during the adipogenic program. A: Relative glycerol release from C3H10T1/2 adipocytes treated with Ad-shLacZ or Ad-shSCD1 for 3 days before the lipolysis experiment. Adipocytes were cultured without or with 0.1 μM CL-316,243 (cell culture medium was changed to phenol red-free medium containing 2% BSA without FAs). The glycerol content of the culture medium was quantified with a glycerol release kit (Applygen, E1002) at 1, 2, and 4 h (n = 4 for each group). Total cellular protein was quantified for the glycerol normalization. Experiments were independently repeated three times. B: Oil Red O staining of C3H10T1/2 cells treated with Ad-shLacZ or Ad-shSCD1. C: Relative mRNA expression of *Atgl* and *Hsl* in cells treated with Ad-shLacZ or Ad-shSCD1 for 3 days (n = 5 in each group). D: Representative Western blot of the lipase proteins ATGL, HSL, and p-HSL^ser660^ in cells treated with Ad-shLacZ or Ad-shSCD1 for 3 days. E: Representative Western blot of the lipophagy marker proteins (p62 and LC3-II) in cells treated with Ad-shLacZ or Ad-shSCD1 for 3 days. F: Relative mRNA expression of the transcriptional regulators and FA oxidation enzymes, nuclear-encoded mitochondrial genes in cells treated with Ad-shLacZ or Ad-shSCD1 for 3 days (n = 5 in each group). Statistical analysis: two-way ANOVA in A, unpaired Student’s *t*-test in C and F. Data were expressed as mean ± SD. *P indicated for the comparisons at the basal condition (Ad-shLacZ vs. Ad-shSCD1); #*P* indicated for the comparisons at the CL-316,243 stimulated condition (Ad-shLacZ vs. Ad-shSCD1). Data were expressed as mean ± SD. *P < 0.05, **P < 0.01, ***P < 0.001. #*P* < 0.05, ##*P* < 0.01, ###*P* < 0.001.

To determine whether the effect of SCD1 on mature adipocytes is reversible, C3H10T1/2 adipocytes with recombinant adenovirus Ad-shSCD1 were used to disrupt SCD1 expression, with Ad-shLacZ as a control. Infecting C3H10T1/2 adipocytes with recombinant adenovirus Ad-shSCD1 effectively downregulated SCD1 protein levels (**Fig. 4D**). SCD1 knockdown significantly reduced glycerol release levels in adipocytes both under basal condition and CL-316,243-stimulated condition, when compared with control group Ad-shLacZ (Fig. 4A). In contrast to SCD1 overexpression, Ad-shSCD1-infected adipocytes had a larger size of LDs (Fig. 4B). Moreover, ATGL and HSL mRNA and protein levels were decreased in SCD1 knockdown adipocytes (Fig. 4C, D). Treated with leupeptin, LC3-II protein levels were decreased, while the p62 protein level increased in SCD1 knockdown adipocytes (Fig. 4E) indicating reduced lipophagy, which is consistent with the above result that SCD1 knockdown reduced lipolysis. The transcriptional regulators, *Pparα* and *Prdm16*, and FA oxidation genes, *Cpt1β*, *Mcad*, and *Ndufa5*, were significantly downregulated by Ad-shSCD1 (Fig. 4F).

### SCD1 promotes fat mobilization in scWAT and increases the whole-body energy expenditure of mice

**Fig. 5.**
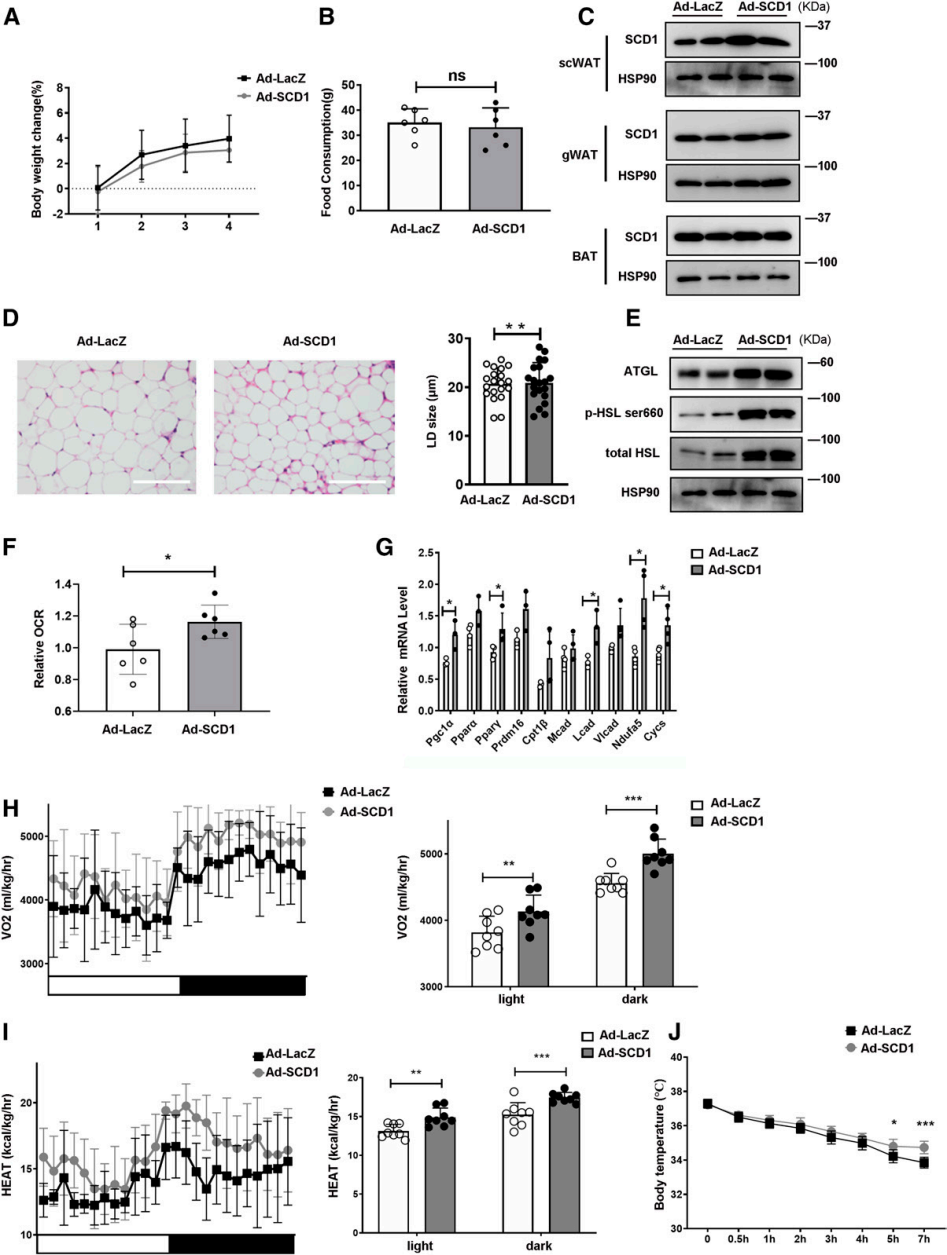
Overexpression of SCD1 promotes fat mobilization and thermogenesis in scWAT and enhances whole-body energy expenditure of mice. A: Body weight change of mice during four adenovirus injections (n = 6 for each group). B: Total food consumption of mice injected with adenovirus for 2 weeks (n = 6 in each group). C: Representative Western blots from scWAT, gWAT, and BAT adipocytes of SCD1. D: H&E staining of scWAT from mice injected with Ad-LacZ or Ad-SCD1 adjacently to the scWAT. E: Representative Western blots from scWAT adipocytes of ATGL and HSL. The same samples from the same mouse scWAT in C and E. F: Basal OCR of scWAT was measured and shown (n = 6 in each group). Data were normalized to the Ad-LacZ group. G: Relative mRNA expression of the transcriptional regulators, FA oxidation enzymes, and nuclear-encoded mitochondrial genes in scWAT treated with Ad-LacZ or Ad-SCD1 (n = 4 in each group). H: Whole-body oxygen consumption rate (VO_2_) of the mice during a 12 h light/12 h dark cycle was measured and the mean of the 12 h light/12 h dark cycle were displayed (n = 8 mice in each group). I: Heat generation of mice in a 12 h light/12 h dark cycle was calculated and the average values for the 12 h light/12 h dark cycle are displayed (n = 8 mice in each group). J: Rectal temperature of mice injected with Ad-LacZ or Ad-SCD1 at 4°C for 7 h (n = 6 for each group). Statistical analysis: unpaired Student’s *t*-test in A, B, D, F, and G; two-way ANOVA in H, I, and J. Data were expressed as mean ± SD. *P < 0.05, **P < 0.01, ***P < 0.001.

To further explore the effect of SCD1 on lipolysis and lipid mobilization in vivo, we infected the scWAT of mice with Ad-SCD1 to upregulate the expression of SCD1 ectopically, while littermates were infected with Ad-LacZ as controls. During the 2 week period of adenovirus injection, we measured the body weight and food intake and found that there were no significant differences between them (**Fig. 5A, B**). Western blot showed that SCD1 expression was effectively increased in scWAT, while gWAT or BAT was not affected (Fig. 5C), indicating a specific overexpression of SCD1 in scWAT. scWAT of Ad-SCD1-treated mice had a smaller LD size than that of Ad-LacZ-treated mice (Fig. 5D). Moreover, ATGL and HSL protein levels were upregulated in the Ad-SCD1 group (Fig. 5E). To better investigate the role of SCD1 in the thermogenesis of scWAT, studies of scWAT respiration were performed. The basal OCR was promoted (Fig. 5F), and the transcriptional regulators, *Pgc1α* and *Pparγ*, and FA oxidation genes, *Lcad*,* Ndufa5*, and *Cycs*, were enhanced in the scWAT of Ad-SCD1-treated mice (Fig. 5G).

Adipocyte mobilization of FA is instrumental for energy expenditure (41). Mobilization of TAG-FA is accompanied by maintenance of core body temperature during cold exposure, and energy balance may also be benefitted (42). Therefore, we attempted to determine the role of SCD1 in whole-body energy metabolism. We used CLAMS analysis to explore the function of SCD1 in oxygen consumption and heat production. Basal oxygen consumption rates (VO_2_) of the Ad-SCD1 group were markedly higher than those of Ad-LacZ group (Fig. 5H). SCD1 overexpression in scWAT significantly increased whole-body energy consumption (Fig. 5I). To further study the differences in energy consumption among these mice, we performed a cold tolerance test to investigate adaptive thermogenesis, another important component of energy consumption (43, 44). During cold exposure, compared with Ad-lacZ-treated mice, the body temperature drops of Ad-SCD1 treated mice were significantly less (Fig. 5J), which indicated that in cold exposure, overexpressing SCD1 in scWAT can improve the body’s adaptability by generating more heat in mice.

### Knockdown of SCD1 inhibits fat mobilization in scWAT and reduces whole-body energy expenditure

**Fig. 6.**
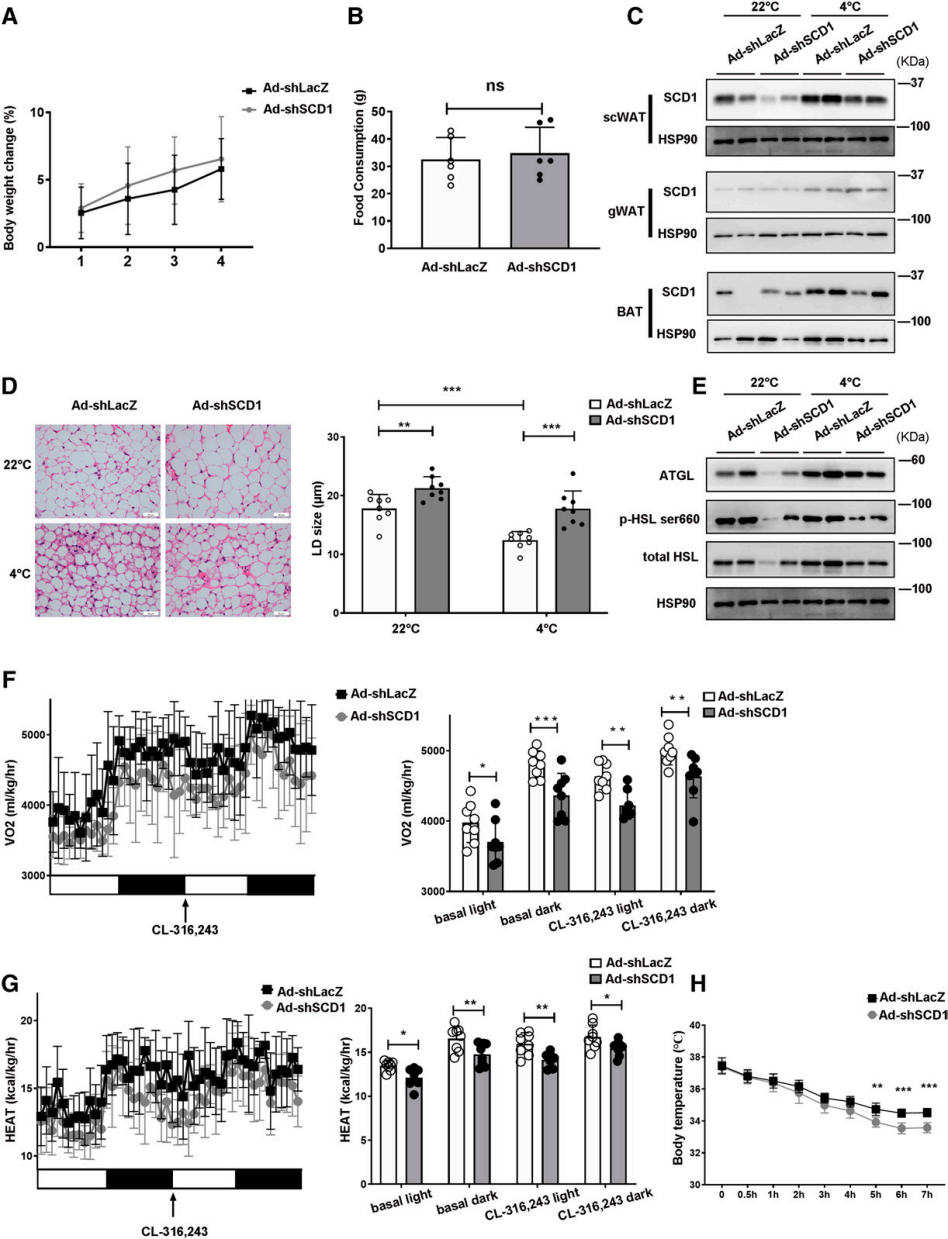
In mice, SCD1 knockdown inhibits fat mobilization in scWAT lipolysis and decreases whole-body energy expenditure. A: Body weight change of mice during four adenovirus injections (n = 6 for each group). B: Total food consumption of mice injected with adenovirus for 2 weeks (n = 6 in each group). C: Representative Western blots from scWAT, gWAT, and BAT adipocytes of SCD1. D: H&E staining of scWAT from mice injected with Ad-shLacZ or Ad-shSCD1 adjacently to the scWAT. E: Representative Western blots from scWAT adipocytes of ATGL and HSL. The same samples from the same mouse scWAT in C and E. F: Whole-body oxygen consumption rate (VO_2_) of the mice in basal and CL-316,243 conditions during a 12 h light/12 h dark cycle was measured and the mean in basal and CL-316,243 conditions of the 12 h light/12 h dark cycle were displayed (n = 8 mice in each group). G: Heat generation of mice in basal and CL-316,243 conditions in a 12 h light/12 h dark cycle was calculated and the average values for the 12 h light/12 h dark cycle in basal and CL-316,243 conditions are displayed (n = 8 mice in each group). H: Rectal temperature of mice injected with Ad-shLacZ or Ad-shSCD1 at 4°C for 7 h (n = 6 for each group). Statistical analysis: unpaired Student’s *t*-test in A, B, and D; two-way ANOVA in F, G. and H. Data were expressed as mean ± SD. *P < 0.05, **P < 0.01, ***P < 0.001.

To further clarify the role of SCD1 in fat mobilization in scWAT adipocytes, we then downregulated SCD1 expression in mouse scWAT by infecting it with recombinant adenovirus expressing Ad-shSCD1, while littermates injected with Ad-shLacZ were used as controls. During the 2 weeks of adenovirus injection, we measured the body weights of the mice and their food intake, and found that there was no significant difference between them (**Fig. 6A, B**). We found that SCD1 knockdown specifically occurred in scWAT, while gWAT or BAT was not affected (Fig. 6C). H&E analysis showed that cold exposure induced scWAT beige adipocyte formation, as indicated by smaller LDs, and this effect was weakened by SCD1 knockdown (Fig. 6D). Consistent with the histological phenotypes, expression ATGL and HSL was upregulated in response to cold in the control mice, while their increase induced by cold was inhibited by SCD1 knockdown (Fig. 6E). It has been reported that SCD1 inhibition promotes adipose tissue inflammation (45), and in 3T3-L1 adipocytes, SA strongly induced the changes of inflammatory gene expression (46). We next examined inflammatory cytokines in the adipose tissue of Ad-shSCD1- or Ad-shLacZ-injected mice. Consistent with previous studies, knockdown of SCD1 in scWAT increased the expression of interleukin (*Il*)-*6*, *Il-1β*, tumor necrosis factor α (*Tnfα*), and interferon γ (*Infγ*) (supplemental Fig. S4). Moreover, SCD1 knockdown mice consumed less oxygen during a 12 h-light and 12 h-dark cycle under basal and injection with the β3-adrenergic agonist CL316,243 conditions (Fig. 6F). SCD1 knockdown also blocked heat generation in mice under basal and CL-316,243-stimulated conditions (Fig. 6G), which was consistent with the impaired adaptive thermogenesis by the knockdown of SCD1 (Fig. 6H). These results suggested that SCD1 knockdown in scWAT inhibited lipid mobilization and reduced the energy expenditure.

### SCD1 promotes lipolysis through its product, OA

**Fig. 7.**
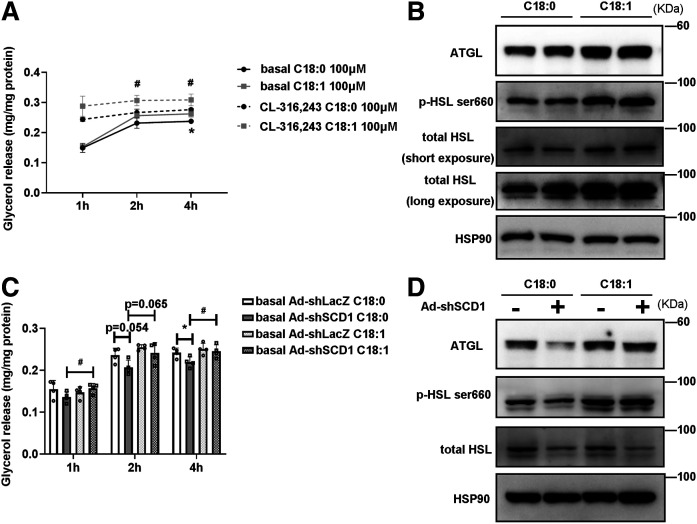
OA promotes lipolysis and enhances lipase expression in differentiated C3H10T1/2 adipocytes. A: Relative release of glycerol from C3H10T1/2 adipocytes treated with C18:0 (SA) or C18:1 (OA) at 100 μM for 24 h before the lipolysis experiment. Adipocytes were cultured without or with 0.1 μM of CL-316,243 (cell culture medium was changed to phenol red-free medium containing 2% BSA without FAs). The glycerol content of culture medium was quantified with a glycerol release kit (Applygen, E1002) at 1, 2, and 4 h (n = 4 for each group). Total cellular protein was quantified for the glycerol normalization. Experiments were independently repeated three times. B: Representative Western blots of the lipases (ATGL, total HSL, and p-HSL^ser660^) in cells treated with C18:0 or C18:1 at 100 μM for 24 h. C: Relative release of glycerol from differentiated C3H10T1/2 adipocytes treated with Ad-shLacZ or Ad-shSCD1 for 2 days and then adding 100 μM C18:0 or 100 μM C18:1 for 24 h before the lipolysis experiment. Cell culture medium was changed to phenol red-free medium containing 2% BSA without FA. The glycerol content of culture medium was quantified with a glycerol release kit (Applygen, E1002) at 1, 2, and 4 h (n = 4 in each group). Total cellular protein was quantified for the glycerol normalization. D: Representative Western blots of the lipases (ATGL, total HSL, and p-HSL^ser660^) in cells treated with Ad-shLacZ or Ad-shSCD1 for 2 days and then adding 100 μM C18:0 or 100 μM C18:1 for 24 h. Statistical analysis: two-way ANOVA in A and C. Data were expressed as mean ± SD. In A, *P indicated for the comparisons at the basal condition (100 μM SA vs. 100 μM OA); #*P* indicated for the comparisons at the CL-316,243-stimulated condition (100 μM SA vs. 100 μM OA). In C, *P indicated for the comparisons at basal C18:0 treatment conditions (Ad-shLacZ vs. Ad-shSCD1); #*P* indicated for the comparisons at the Ad-shSCD1 treatment conditions (100 μM C18:0 vs. 100 μM C18:1). *P < 0.05, **P < 0.01, ***P < 0.001. #*P* < 0.05, ##*P* < 0.01, ###*P* < 0.001.

We have shown that the percentage of TAG MUFAs, especially OA, in scWAT adipocytes was increased after cold exposure (Fig. 1A, B). However, whether SCD1 promotes lipolysis through its product OA is unknown. To investigate whether TAG metabolism in adipocytes is regulated by OA, differentiated C3H10T1/2 cells were treated with OA or SCD1 substrate, SA (C18:0), as control. Then lipolysis was evaluated 24 h later. FA doses between 50 μM and 500 μM are considered to be within the physiologically relevant range of human and rodent models (46–49). According to previous studies, we chose FA doses of 100 and 500 μM. Lipolysis was significantly increased by treatment with OA at a dose of 100 μM, which can be evidenced by the fact that OA-treated cells released glycerol into medium more easily than SA-treated cells at that dose (**Fig. 7A**). Furthermore, the increase in lipolysis induced by OA rather than SA was related to a significant increase in the protein content of ATGL and HSL Fig. 7B.

Next, we performed experiments to evaluate whether SCD1 enhanced lipolysis through its product, OA. In adipocytes, we inhibited SCD1 with Ad-shSCD1 and then treated them with 100 μM OA or 100 μM SA. As shown in Fig. 4A, knockdown of SCD1 inhibited the glycerol release levels in cultured adipocytes. However, after treating the cells with OA, the role of Ad-shSCD1 in reducing glycerol release was blunted, as compared with the control group in which SA was used to treat the cells (Fig. 7C). Similarly, Ad-shSCD1 reduced lipase protein expression (Fig. 4D); however, after treating Ad-shSCD1 adipocytes with 100 μM of OA, the effects of Ad-shSCD1 on downregulating lipase proteins were attenuated (Fig. 7D). We repeated this experiment using 500 μM of OA or SA. We observed that lipolysis was also activated in C3H10T1/2 adipocytes treated with 500 μM of OA but not SA (supplemental Fig. S5). These results indicated that the actions of SCD1 on gene expression linked to lipases and lipid mobilization are dependent on OA.

### SCD1 is regulated by BMP4 via the Smad signaling pathway

**Fig. 8.**
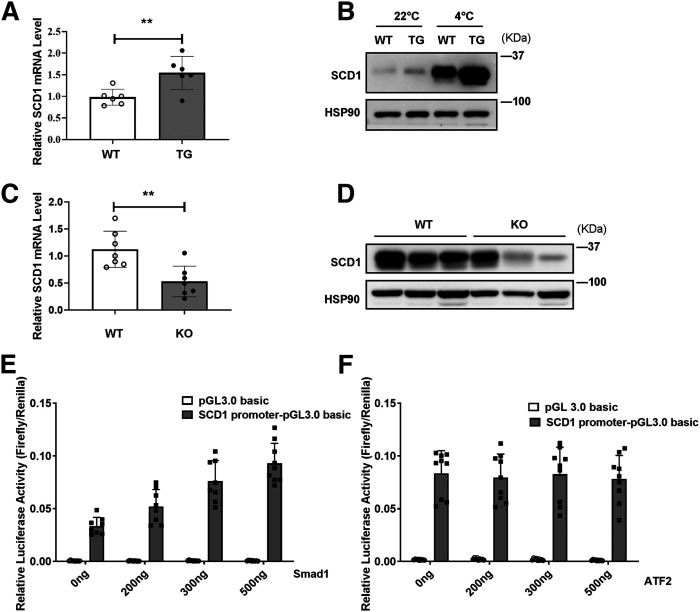
BMP4 participates in promotion of SCD1 expression. A: Relative mRNA expression of *Scd1* in scWAT adipocytes of WT and BMP4 TG mice housed at 22°C. B: Representative Western blots of SCD1 protein expression in scWAT adipocytes of WT and BMP4 TG mice housed at RT (22°C) or during cold exposure (4°C) for 3 days. C: Relative mRNA expression of *Scd1* in scWAT adipocytes of control group (WT) and Fabp4-Cre-Bmp4^LoxP/LoxP^ (KO) mice housed at RT. D: Representative Western blots of SCD1 protein expression in scWAT adipocytes of WT and BMP4 KO mice housed at RT. E: Luciferase assays for SCD1 promoter constructs and Smad1 transfected into 293T cells. F: Luciferase assays for SCD1 promoter constructs and ATF2 transfected into 293T cells. Statistical analysis: unpaired Student’s *t*-test in A and C. Data were expressed as mean ± SD. *P < 0.05, **P < 0.01, ***P < 0.001.

Our recent studies reported that mice with BMP4 specifically overexpressed in adipocytes showed a decrease in the size of white adipocytes along with an increase in the number of beige adipocytes. These changes are closely related to increased energy expenditure (36). All the phenotypes of scWAT in BMP4 TG mice are similar to the scWAT in SCD1-overexpressing mice. As a result, we assessed whether BMP4 regulates SCD1 to promote energy expenditure. We first tested the *Scd1* mRNA level in WT and BMP4 TG mice at RT (22°C). We found that the *Scd1* mRNA level was increased in the scWAT of BMP4 TG mice (**Fig. 8A**). Then we explored the SCD1 protein level in WT and BMP4 TG mice at RT (22°C) and during cold exposure (4°C) for 3 days. Interestingly, we found that SCD1 was upregulated in BMP4 TG mice, especially after cold exposure (4°C) for 3 days (Fig. 8B). In mice with adipose tissue BMP4 KO (Fabp4-Cre-Bmp4^LoxP/LoxP^), the scWAT showed lower mRNA and protein levels of SCD1 than the control group (Fig. 8C, D).

Previous studies have shown that the mothers against decapentaplegic homolog (Smad) and p38/mitogen-activated protein kinase are two important signaling pathways downstream of BMPs (36). We examined the role of Smad1 and activating transcription factor 2 (ATF2) (ATF2 is located downstream of p38/mitogen-activated protein kinase) in regulating the expression of SCD1. A luciferase reporter assay showed that overexpression of Smad1 (Fig. 8E), not ATF2 (Fig. 8F), significantly enhanced SCD1 promoter activity, indicating that Smad1 may act on the upstream of SCD1 to regulate its mRNA expression. Together these findings showed that BMP4 is one of the upstream factors of SCD1, and BMP4 upregulates SCD1 via the Smad signaling pathway.

**Fig. 9.**
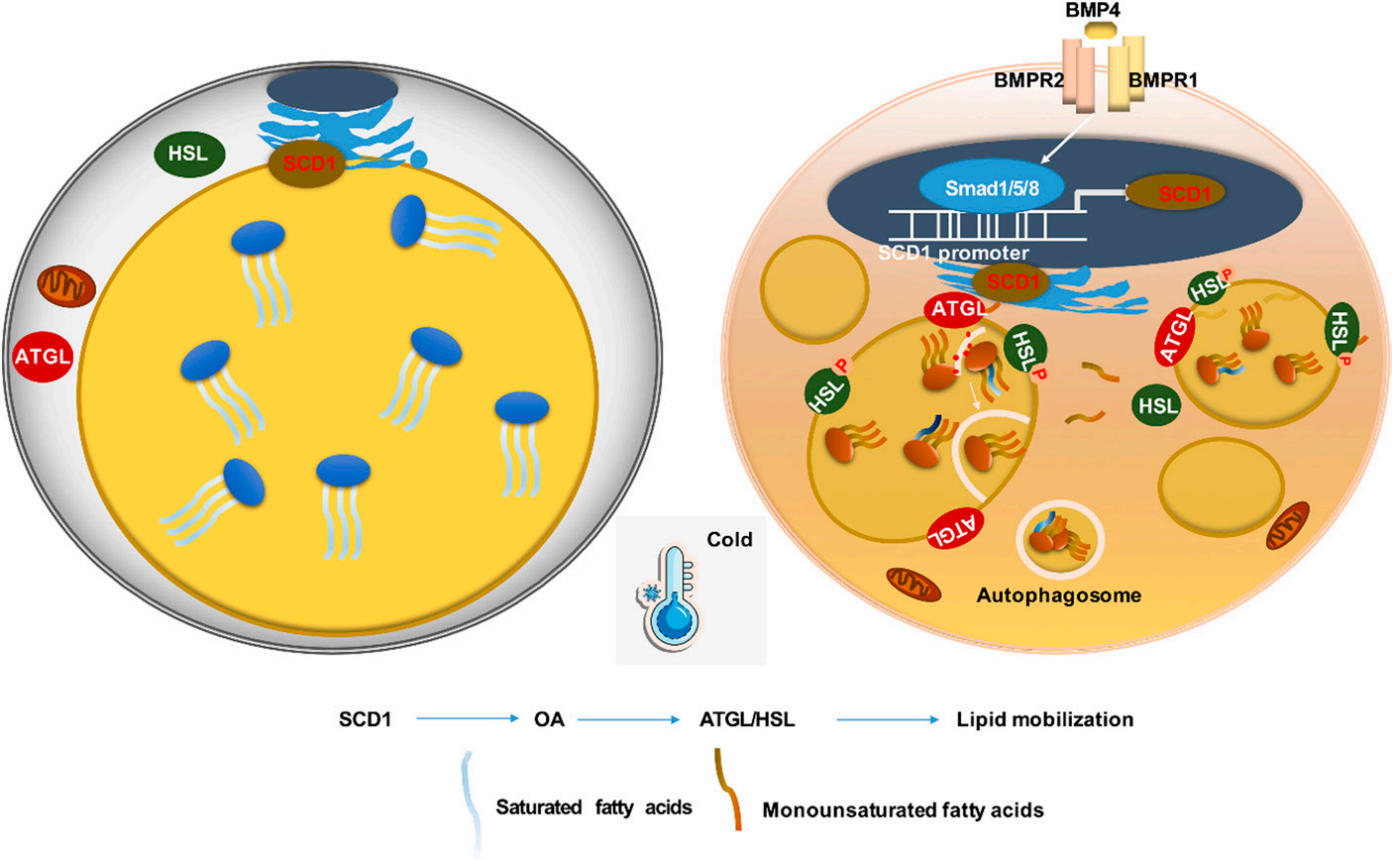
Schematic model highlights the SCD1 function on adipocytes. SCD1 is highly expressed on the endoplasmic reticulum of mature adipocytes isolated from scWAT and catalyzed the desaturation of long-chain SFAs to MUFAs. When exposed to cold or BMP signaling stimulation, SCD1 expression on adipocytes increases and produces more MUFAs, which triggers the browning change in adipocytes. On top of that, one of SCD1 products, OA (C18:1), caused the activation of lipid mobilization, including lipolysis and lipophagy, and further promoted thermogenesis. In conclusion, SCD1 is a new and important regulator of adipocyte lipid mobilization and energy homeostasis.

## DISCUSSION

SCD1 is a microsomal enzyme involved in MUFA biosynthesis, mainly oleic acid and palmitoleic acid. Previous studies reported that SCD1 KO mice exposed to cold (4°C) exhibited hypothermia (28). Another study showed that loss of UCP1 not only promoted inguinal WAT lipolysis but also elevated SCD (29). All of these studies demonstrated that SCD1 gene expression plays an important role in lipid metabolism and thermal regulation; however, the mechanism of SCD1 in regulating fat mobilization is currently unknown.

In this study, we demonstrated that under cold exposure, the SCD desaturation index and all four SCD isoforms were upregulated, while SCD1 showed the largest fold change (Figs. 1I, 2) in scWAT adipocytes of mice. As a result, the percentage of total MUFAs, especially OA, of TAG in scWAT adipocytes was significantly elevated after cold exposure (Fig. 1). By using the C3H10T1/2 adipocyte model, we demonstrated that SCD1, as a lipogenic gene, promoted lipid mobilization by enhancing lipolysis and lipogenesis. SCD1 promoted lipolysis through upregulated ATGL and HSL expression and enhanced lipophagy. As a result, more lipases were recruited around LDs to promote lipolysis under β3-aderenergic stimulation (Fig. 3). It has been reported that the protein abundance of LC3-II was induced and p62 levels were reduced after ATGL overexpression, suggesting increased autophagy (14). In our study, we found that SCD1 overexpression increased protein and mRNA levels of ATGL, upregulated the LC3-II protein level, and downregulated the p62 level. We supposed that SCD1 promotes lipophagy through regulating ATGL levels. Moreover, in vivo studies showed that SCD1 plays a potential role in the thermogenesis effects of cold-induced scWAT, as indicated by the results that SCD1 overexpression in scWAT promoted lipolysis and thermogenesis in scWAT, increased energy expenditure, and stabilized body temperature; while knockdown of SCD1 inhibited lipolysis, decreased energy expenditure, and lowered temperature (Figs. 5, 6). The product of SCD1, OA, promoted lipolysis through upregulating lipases (Fig. 7). In addition, we detected that SCD1 is upregulated by BMP4 via the Smad signaling pathway (Fig. 8). The upregulation of SCD1 by BMP4 and its consequence of promoting lipid mobilization may contribute to the enhanced metabolic activity in BMP4 transgenic mice. In conclusion, the findings of our present study suggested an important role for SCD1 in lipid mobilization and adaptive thermogenesis (**Fig. 9**).

Typical white adipocytes possess a large TAG-rich LD that stores excess energy (50). Based on the mounting evidence of WAT browning so far (51–56), it seems that beige adipocytes can promote whole-body heat production through several UCP1-dependent and non-UCP1-dependent mechanisms. These include creatine /phosphocreatine cycling and activation of the ATP-dependent Ca^2+^ cycling through sarco/endoplasmic reticulum Ca^2+^-ATPase 2b and ryanodine receptor 2 and TAG hydrolysis/lipogenesis (6). It has been reported in humans that cold stress increased lipolysis, FFA rate of turnover, and TAG/FFA cycling; the results further emphasized the importance of the TAG/FFA cycle in amplifying the ability of stored TAG to respond quickly to significant changes in energy consumption due to continuous cold stress (57). Enhanced TAG breakdown and resynthesis of beige adipocytes may be an important thermogenic mechanism under cold acclimation conditions (58).

Previous studies demonstrated that acyl-CoA:diacylglycerol acyltransferase 2 (DGAT2) catalyzed the final reaction to synthesize TAG and were located very close to SCD1. These indicated preference for endogenous MUFAs in TAG synthesis (59). The level of desaturase activity was positively correlated with TAG accumulation, which suggested that endogenous UFAs promote TAG storage (60). All these studies demonstrated that SCD1 is important for FA esterification and TAG synthesis. In our study, we found that SCD1 was able to promote mobilization of TAG by upregulating ATGL and HSL. Therefore, simultaneous reactions of TAG hydrolysis and resynthesis exist a dynamic cycle in adipocytes, which is able to be accelerated by SCD1. These results together with our present data suggest that SCD1 plays an important role in regulating lipid remodeling for maintaining a high rate of TAG lipolysis/re-esterification in beige adipocytes. In addition, our results supported the potential of SCD1 in scWAT as a target that promotes dynamic lipid circulation and is beneficial to systemic metabolism. Some limitations exist in this study. The mechanism of OA that regulates lipases is still unknown. In addition, besides OA, some unexplored MUFAs may also be involved in SCD1 promoting lipolysis. More studies and further exploration are needed in the future.

### Data availability

All data pertaining to the findings of this study are available upon request from the corresponding author.

## Supplementary Material

Supplemental Data
