# Supplementary material for: SCD1 promotes lipid mobilization in subcutaneous white adipose tissue
Source: J Lipid Res. 2020 Sep 25;61(12):1589–604. doi: 10.1194/jlr.RA120000869 (PMC7707166; doi:10.1194/jlr.RA120000869)
Supplement: Supplemental Data [file supp_61_12_1589__index.html]

SCD1 Promotes Lipid Mobilization in Subcutaneous White Adipose Tissue — Regulation of lipid mobilization by SCD1 — SCD1 promotes lipid mobilization in subcutaneous white adipose tissue — Supplemental Data 

# SCD1 promotes lipid mobilization in subcutaneous white adipose tissue

## Supplemental Data

- SCD1 Promotes Lipid Mobilization in Subcutaneous White Adipose Tissue - Supplemental Data
