# Supplementary material for: SCD1 promotes lipid mobilization in subcutaneous white adipose tissue
Source: J Lipid Res. 2020 Sep 25;61(12):1589–604. doi: 10.1194/jlr.RA120000869 (PMC7707166; doi:10.1194/jlr.RA120000869)
Supplement: Supplemental Data [file supp_RA120000869_160499_1_supp_595993_qgwb90.pdf]

**SUPPLEMENTAL INFORMATION:**

# SCD1 Promotes Lipid Mobilization in Subcutaneous White Adipose Tissue

Ying Zou,[1,*] Yi-Na Wang,[1,*] Hong Ma,† Zhi-Hui He,* Yan Tang,* Liang Guo,* Yang Liu,* Meng Ding,* Shu-Wen Qian,[2,*] Qi-Qun Tang[2,*]

*Key Laboratory of Metabolism and Molecular Medicine, the Ministry of Education; Department of Biochemistry and Molecular Biology of School of Basic Medical Sciences and Department of Endocrinology and Metabolism of Zhongshan Hospital, Fudan University, Shanghai Medical College, Shanghai 200032, China

†Key Laboratory of Metabolism and Molecular Medicine, the Ministry of Education; Institutes of Biomedical Sciences, Fudan University, Shanghai 200032, China.

**Supplemental Figure S1**

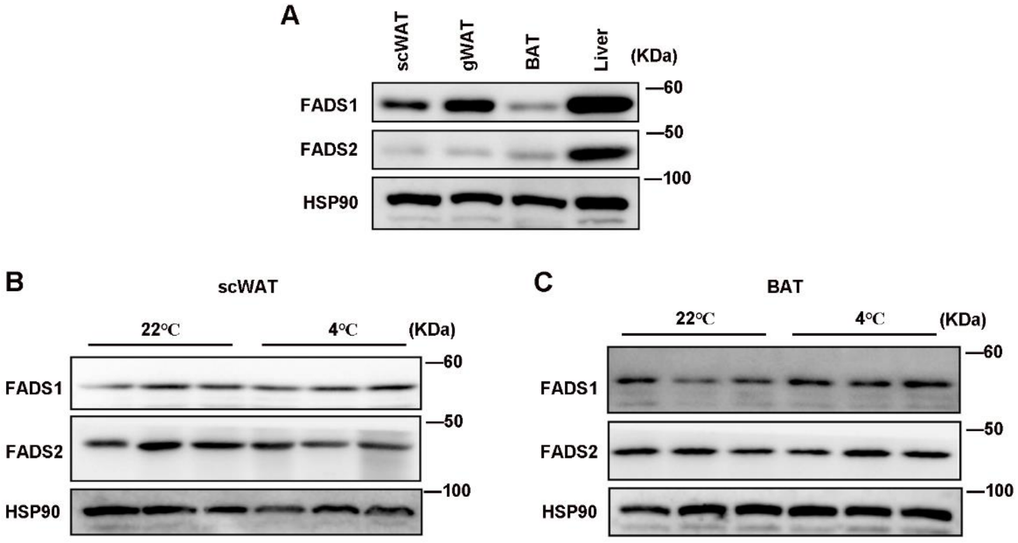


**Supplemental Fig. S1: FADS1, FADS2 are enriched in liver, and have no change after cold exposure in adipocytes. (**A) Tissue distribution of FADS1 and FADS2 protein expression in adipocytes of scWAT, gWAT, BAT and liver from the same mice of Figure 2A. **(**B) Representative western blot of FADS1 and FADS2 expression in scWAT adipocytes from the same mice of Figure 2B housed at 22°C or 4°C for 72h (n=3 for each group). **(**C) Representative western blot of FADS1 and FADS2 expression in BAT adipocytes from the same mice of Figure 2C housed at 22°C or 4°C for 72h (n=3 for each group). Figure 2A and Figure S1A were performed with the same sample, so they have the same HSP90 for loading control. The same HSP90 for loading control is also applied to Figure 2B and Figure S1B, Figure 2C and Figure S1C.

**Supplemental Figure S2**

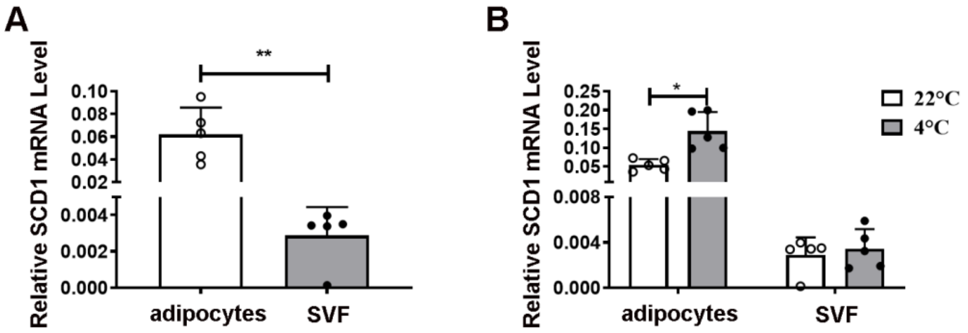


**Supplemental Fig. S2: *Scd1* is enriched in mature adipocytes and induced in mature adipocytes after cold exposure, but not SVF.** (A) Relative mRNA expression of *Scd1* in mature adipocytes and SVF isolated from scWAT of mice housed at 22°C (n=5 for each group). (B) Relative mRNA expression of *Scd1* in mature adipocytes and SVF isolated from scWAT of mice housed at 22°C and 4°C 3 days (n=5 for each group). Statistical analysis: unpaired student t-test in (A-B). Data were expressed as means ± SD. *P < 0.05, **P < 0.01, ***P < 0.001.

## Supplemental Figure S3

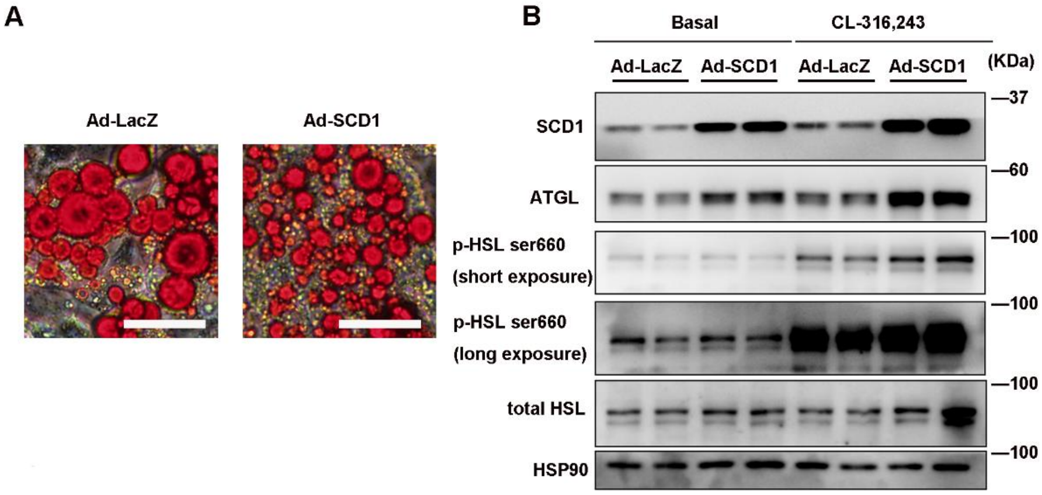


**Supplemental Fig. S3: SCD1 promote lipolysis and this effect is further stimulated by β3-adrenergic receptor signaling.** (A) Oil red O staining of day 11 differentiated C3H10T1/2 mature adipocytes cells, which treated with Ad-LacZ or Ad-SCD1 on day 8 for 3 days. (B) Representative western blot of the lipases protein, ATGL, HSL and p-HSL$^{ser660}$ in cells treated with Ad-LacZ or Ad-SCD1 for 3 days under basal and CL-316,243 stimulated conditions.

**Supplemental Figure S4**

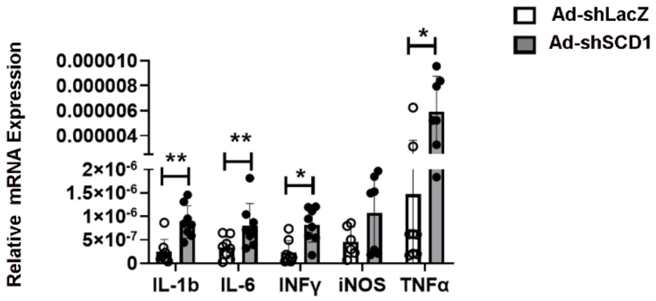


**Supplemental Fig. S4: SCD1 knockdown in scWAT leads to adipose tissue inflammation.** Relative mRNA expression of inflammatory cytokines *Il-1β, Il-6, Infγ, iNOS* and *Tnfα* in scWAT from mice treated with Ad-shLacZ or Ad-shSCD1 (n=6 in each group). Statistical analysis: unpaired student t-test. Data were expressed as means ± SD. *P < 0.05, **P < 0.01, ***P < 0.001.

**Supplemental Figure S5**

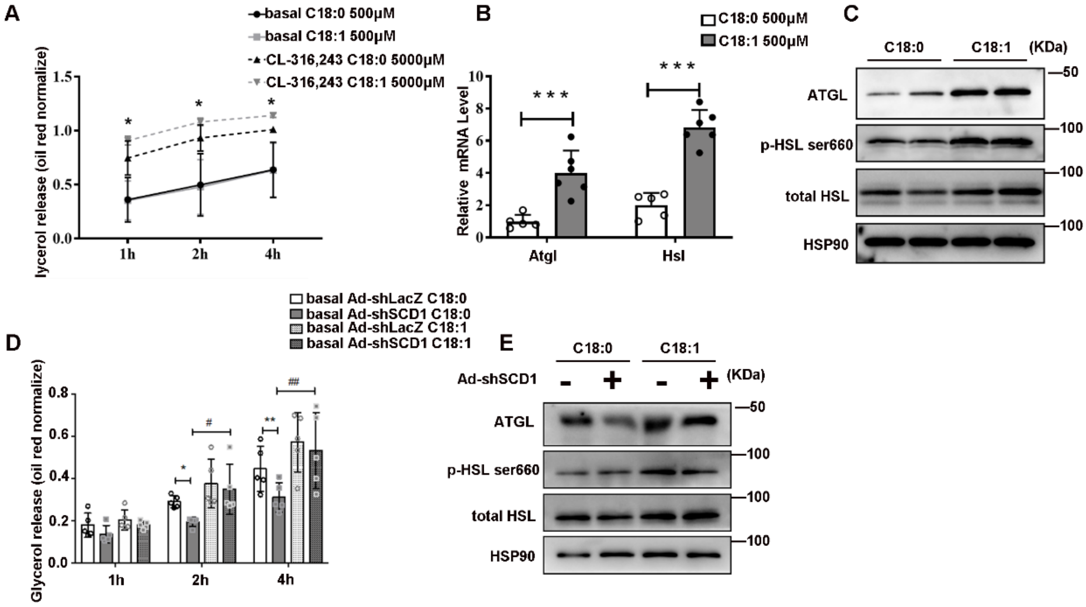


**Supplemental Fig. S5: Oleic acid promotes lipolysis and enhance lipases expression in differentiated C3H10T1/2 adipocytes.** (A) Relative glycerol release from C3H10T1/2 adipocytes, treated with SA or OA at 500 µM for 24 h before the lipolysis experiment. Adipocytes were further subjected without or with 0.1µM CL-316,243 (cell culture medium was changed into phenol red-free medium supplied with 2% fatty acid- free BSA). The glycerol content in culture medium was quantified at 1h, 2h and 4h with Glycerol Release Kit (Applygen, E1002) (n=3 in each group). Oil red O content was quantified for the glycerol normalization. Experiments were repeated three times independently. (B) Relative mRNA expression of the lipases (n=6 for each group). (C) Representative western blot of the lipases (ATGL, total HSL and p-HSL$^{ser660}$) in cells treated with oleic or stearic acids at 500 µM for 24 h. (D) Relative glycerol release from differentiated C3H10T1/2 adipocytes, treated with Ad-shLacZ or Ad-shSCD1 for 2 days then added SA or OA 500 µM for 24 hours before the lipolysis experiment. Cell culture medium was changed into phenol red-free medium supplied with 2% fatty acid- free BSA. The glycerol content in culture medium was quantified at 1h,

2h and 4h with Glycerol Release Kit (Applygen, E1002), and normalized with oil red O quantification at the basal level (n=6 in each group).

Statistical analysis: two-way ANOVA in A and D, unpaired student t-test in B. Data were expressed as means ± SD. In (A) * P indicated for the comparisons at the CL-316,243 stimulated condition (500 μM SA vs. 500 μM OA). In (C) *P indicated for the comparisons at basal C18:0 treatment conditions (Ad-shLacZ vs. Ad-shSCD1); #P indicated for the comparisons at the Ad-shSCD1 treatment conditions (500 μM C18:0 vs. 500 μM C18:1). *P < 0.05, **P < 0.01, ***P<0.001. #P < 0.05, ##P < 0.01, ###P < 0.001.

**Supplemental Table S1**

SCD1 F：TCTCAGAAACACACGCCGACC

SCD1 R：AGGCCGGGCTTGTAGTACCTC

SCD2 F：TGCCTTGTATGTTCTGTGGC

SCD2 R：TCCTGCAAGCTCTACACCTG

SCD3 F：GCCTTGTACGTTCTGTGGCT

SCD3 R：CCCTCCTGCAAGCTCTACAC

SCD4 F: GCCCACTTGCCACAAGAGAT

SCD4 R：GTAGCTGGGGTCATACAGATCA

CPT1β F：GCACACCAGGCAGTAGCTTT

CPT1β R：CAGGAGTTGATTCCAGACAGGTA

PPARα F：AACATCGAGTGTCGAATATGTGG

PPARα R：CCGAATAGTTCGCCGAAAGAA

PPARγ F：AGAACCTGCATCTCCACCTTA

PPARγ R：CCCACAGACTCGGCACTCAAT

ATGL F：GGAGACCAAGTGGAACATCTCA

ATGL R：AATAATGTTGGCACCTGCTTCA

HSL F：TGTGGCACAGACCTCTAAAT

HSL R：GGCATATCCGCTCTC

PGC1α F：GGAGCCGTGACCACTGACA

PGC1α R：TGGTTTGCTGCATGGTTCTG

Cycs F：CCAAATCTCCACGGTCTGTT

Cycs R：CCAGGTGATGCCTTTGTTCT

Ndufa5 F：ATCACCTTCGAGAAGCTGGA

Ndufa5 R：ACTTCACCACCCTGAAGCAA

VLCAD F：GAATGACCCTGCCAAGAACGA

VLCAD R：ATGCCCACAATCTCTGCCAAG

LCAD F：GGACTCCGGTTCTGCTTCCA

LCAD R：TGCAATCGGGTACTCCCACA

MCAD F：CAACACTCGAAAGCGGCTCA

MCAD R：ACTTGCGGGCAGTTGCTTG

18S F：CGCCGCTAGAGGTGAAATTCT

18S R：CATTCTTGGCAAATGCTTTCG
